# Unlocking the Power of Physical Activity in Inflammatory Bowel Disease: A Comprehensive Review

**DOI:** 10.1155/grp/7138811

**Published:** 2024-12-28

**Authors:** Jiajia Chen, Shaopeng Sun

**Affiliations:** ^1^Department of Anesthesiology & Department of Nursing, The First Affiliated Hospital of Zhejiang Chinese Medical University (Zhejiang Provincial Hospital of Traditional Chinese Medicine), Hangzhou, China; ^2^Department of Gastroenterology, The First Affiliated Hospital of Zhejiang Chinese Medical University (Zhejiang Provincial Hospital of Traditional Chinese Medicine), Hangzhou, China

**Keywords:** clinical outcome, inflammatory bowel disease, physical activity, quality of life

## Abstract

**Purpose of Review:** This study reviewed the concept and assessment tools of physical activity (PA), the level and limiting factors of PA in people with inflammatory bowel disease (IBD), and its impact on patient clinical outcomes, aimed at providing a reference for exercise-assisted treatment of people with IBD.

**Recent Findings:** The current findings of PA in patients with IBD focus on the risk of disease, promoting and limiting factors, and the effect of clinical outcomes. Patients with IBD have inadequate levels of PA, and the association of PA with IBD incidence and disease activity remains controversial. Nevertheless, PA has demonstrated beneficial effects on clinical outcomes, particularly in reducing mortality, enhancing quality of life, and improving body composition.

**Summary:** IBD is a chronic disease with no cure. Although medication is the main treatment modality, it requires careful consideration of its risks and benefits. PA has proven to be an effective nondrug treatment that can slow the progression of various chronic diseases and enhance patients' quality of life. However, the correlation between PA levels and clinical outcomes of IBD remains unclear.

## 1. Introduction

Inflammatory bowel disease (IBD) is a chronic nonspecific disease that includes Crohn's disease (CD) and ulcerative colitis (UC). Common clinical symptoms include recurrent abdominal pain, diarrhea, and posterior tenesmus [1, 2]. A primary aim in treating IBD is to enhance the quality of life, given its chronic nature involving alternating between the active and remission phases, requiring lifelong management. The incidence rate of IBD is high in developed countries such as North America and Western Europe. The annual incidence rate can reach 29.3/100,000 people [3]. However, in the past 20 years, the incidence of IBD in Western countries has remained relatively stable or even decreased, while the incidence in Eastern Europe and Asia has shown a continuous increasing trend [4]. More specifically, the incidence of IBD has increased drastically in East Asian countries such as China [5], South Korea [6], and Japan [7].

The course of IBD is characterized by alternating active and remission phases, protracted recovery, and the need for lifelong treatment, all of which seriously affect the patient's quality of life. The etiology and pathogenesis of this disease remain unclear but are generally believed to be closely related to genetics, the environment, etc. Currently, drug therapy is the most crucial treatment approach; however, it encounters numerous challenges, such as low response rates and potential side effects. Increasing research attention is directed toward investigating the potential advantages of nonpharmacological interventions, such as physical activity (PA), for patients with IBD.

Some evidence based on the population shows that the implementation of an exercise intervention can reduce the risk of various chronic diseases, including cancer [8] and cardiovascular disease [9]. Furthermore, exercise interventions can improve patient outcomes with depression [10]. The impact of exercise and PA on the gastrointestinal tract is an emerging research field. Several studies have shown an inverse relationship between PA and the risk of gastrointestinal related diseases such as colon cancer [11], diverticular disease [12], gallstones [13], or constipation [14]. However, some studies have also shown the harm of exercise to gastrointestinal symptoms [15] such as nausea, heartburn, diarrhea, and gastrointestinal bleeding. In recent years, studies have found that exercise may have multiple health benefits for IBD [16–18]. From the perspective of pathological and physiological mechanisms, regular exercise can reduce visceral fat content, inhibit the release of inflammatory factors, and induce an anti-inflammatory microenvironment, providing theoretical support for exercise assisted treatment of IBD [19]. Recent studies have confirmed the existence of the “gut-muscle” axis [20, 21], indicating that exercise has a regulatory effect on gut microbiota, which is crucial in the development of IBD [22].

According to the WHO, engaging in PA can lower the risk of disease progression or premature death while also enhancing physical function and overall quality of life in adults with chronic diseases [23]. Nevertheless, research on PA in patients with IBD is still in its preliminary stages [24]. Given the crucial role of PA, this study reviews the current status of PA in patients with IBD with the aim of providing a reference for PA practice.

## 2. Concept and Assessment Tools for PA

### 2.1. Concepts

PA involves increased energy expenditure of the body resulting from skeletal muscle contraction, including all types of sports or exercise, such as leisure activities and commuting. PA can be categorized into various types, such as static exercise and dynamic exercise, based on the form of muscle contraction. In addition, PA can be classified into anaerobic exercise and aerobic exercise according to the metabolic law of energy supply during exercise. Based on the background of daily life sources, PA can be categorized into occupational, family (household chores), transportation (such as walking and cycling), and leisure (various sports) [25]. The intensity of these activities directly affects the effectiveness and safety of PA for patients with IBD. Key indicators of activity intensity include reserve oxygen uptake, reserve heart rate, oxygen uptake, heart rate, and metabolic equivalent (MET) [26]. In particular, MET is a globally recognized and widely used approach for quantifying the energy expenditure of PA. One MET is the resting metabolic rate, indicating the amount of oxygen consumed while resting quietly in a chair, approximately 3.5 mL/kg/min. The resting state is 0–1.5 METs, low-intensity PA (such as walking, laundry, and cooking) is 1.5–2.9 METs, moderate-intensity PA (such as brisk walking, heavy cleaning work, and dancing) is 3–5.9 METs, and high-intensity PA (such as running, doing heavy agricultural work, and kicking) is 6 METs or higher [27]. We summarized the exercise modes corresponding to PA of different intensities (Table 1). Patients with IBD should perform PA according to their condition and appropriate intensity levels. Studies have indicated that walking for more than 1.5 h per day for patients with IBD does not lead to further improvements in quality-of-life scores. Thus, additional research is needed to determine the optimal intensity of PA that maximizes benefits for patients with IBD. Currently, consideration can be given to adhering to the 2020 WHO guidelines for PA and sedentary behavior [23]. The guide recommended that adults and patients with chronic disease engage in 150–300 min of moderate-intensity or 75–150 min of high-intensity aerobic exercise per week or an equivalent combination of the two.

### 2.2. Assessment Tools

PA can be measured using two primary approaches: objective and subjective. Objective approaches involve the use of wearable devices such as pedometers, accelerometers, and heart rate monitors [28]. Subjective approaches primarily assess the type, frequency, and duration of PA through self-reported questionnaires, scales, or logs. Examples include the International Physical Activity Questionnaire (IPAQ) [29], the Global Physical Activity Questionnaire [30], the Short Questionnaire to Assess Health Enhancing Physical Activity [31], and the Gordon Leisure Time Questionnaire [32]. Among them, the IPAQ has high reliability and validity and has been extensively employed in numerous countries [29]. The IPAQ can be divided into two versions: long and short. The long version comprises five sections: occupation, household chores, transportation, leisure PA, and sitting. Regarding the frequency and time of PA inquiries in the PA project, each PA intensity was truncated at 180 min. The short test comprises seven questions that assess the total time individuals spend each week in four types of PA: intense PA, moderate PA, walking, and sitting. The IPAQ recommends computing an individual's weekly PA level (MET minute per week). This is done by multiplying the MET value of a specific intensity of PA by the average daily activity time (minute per day) and the number of days per week the activity is performed. The total weekly PA level was then determined by summing the PA levels at different intensities.

## 3. PA and IBD Incidence Risk

Epidemiological data indicate that the incidence rate of IBD is relatively high in Europe (505 per 100,000 people), Canada (248 per 100,000 people), and the United States (214 per 100,000 people), among other developed regions [3]. In addition, the incidence rate of IBD annually increases in developing countries and regions, such as Asia, the Middle East, and South America [4]. The etiology of IBD remains unclear, and increasing research is examining the influence of lifestyle factors on IBD onset. The correlation between PA as a lifestyle and IBD onset remains a topic of debate.

First, some studies revealed a correlation between the incidence of IBD and occupational characteristics, possibly because of the varying PA levels among different occupational groups. Sonnenberg [33] analyzed the occupational distribution of 12,014 patients with IBD using German social security statistics and discovered that outdoor work and high PA level work were protective factors for IBD onset. Conversely, occupations involving working in air-conditioned environments or irregular shifts for extended periods were associated with an increased risk of IBD. In a prospective study in Denmark, researchers conducted a 5-year or 10-year follow-up survey involving over 2.2 million people, and the findings indicated that sedentary work may increase the risk of developing IBD [34].

Second, there is a relationship between IBD risk and PA levels. Persson et al. [35] conducted a case–control study examining various risk indicators associated with IBD. In an analysis involving 152 patients with CD, 145 patients with UC, and 305 control populations, regular weekly relative ratio (RR) = 0.6, 95% confidence interval (CI): 0.4–0.9) and daily (RR = 0.5) PAs (95% CI: 0.3–0.9) were found to potentially reduce the risk of developing CD. In a case–control study [36] conducted in Slovakia involving 338 adult patients with IBD (190 with CD and 148 with UC), insufficient PA during childhood was associated with an increased incidence of CD (odds ratio (OR) = 2.7, 95% CI: 1.5–5.0) and UC (OR = 2.0, 95% CI: 1.1–3.5). Insufficient PA level was identified as an independent risk factor for IBD onset [36]. In a national health interview survey [37] conducted in the United States with 60,155 participants, 786 individuals (1.3%) had IBD. The study revealed a positive correlation between IBD incidence and PA deficiency (OR = 1.38, 95% CI: 1.16–1.66) [37]. Klein et al. [38] compared environmental factors between newly diagnosed patients with IBD and healthy controls and found that the control group tended to engage in moderate or severe PA before bedtime, whereas patients with IBD reported less PA before bedtime. Furthermore, one extensive prospective cohort study [39] focusing on American women identified a negative relationship between PA and the incidence of CD, but not UC.

However, some studies have reported that the incidence of IBD is not correlated with PA levels. For instance, the European Cancer and Nutrition Prospective Study cohort assessed and monitored 300,724 participants, identifying 177 confirmed cases of UC and 75 cases of CD [40]. No significant correlation was observed between PA and UC (*p* = 0.97) or CD (*p* = 0.42). Furthermore, Halfvarson et al. [41] examined the potential exposure factors for IBD in a genetically identical Swedish–Danish twin cohort and effectively mitigated the potential influence of genetic factors. Their findings revealed no significant difference in PA between the patients and their twins before the diagnosis of IBD [41].

In summary, research findings on the causal relationship between IBD incidence and PA levels are inconsistent (Table 2). This variability could stem from different confounding factors and retrospective biases. Future large-scale studies are necessary to provide more definitive evidence and confirm this causal relationship.

## 4. Promoting and Limiting Factors of PA in Patients With IBD

Although many patients with IBD recognize the positive impact of PA on their condition and are willing to engage in it, their PA levels are generally lower due to symptoms such as abdominal discomfort, fatigue, pain, negative emotions, and other related factors. Based on a survey conducted by Guthold et al. across 51 countries, 17.7% of healthy adults exhibit insufficient levels of PA [42]. The proportion of patients with IBD with insufficient PA is significantly higher than that of healthy adults. Moreover, there is no substantial difference in PA levels between active and remission patients with CD [43]. Tew, Jones, and Mikocka-Walus reported that 33.3% of patients with IBD do not meet the recommended PA levels [44]. Conversely, Fagan, Osborne, and Schult found that only 66% of patients with IBD in remission or with mild-to-moderate activity met the WHO recommendation of at least 150 min of moderate-intensity aerobic PA per week [45]. Furthermore, patients with IBD typically engage in low-intensity PA. In a survey involving 859 adult patients with IBD conducted by Tew, Jones, and Mikocka-Walus [44], 57% reported walking as their most common activity, 34% expressed a preference to avoid running or jogging, and only 17% reported participating in high-intensity PA. Thus, when advising patients with IBD about physical activities, it is crucial to consider their preferences and prioritize low-intensity activities to encourage their full participation and enthusiasm.

Various factors limit patients with IBD from participating in PA, and effectively identifying these factors can aid in developing targeted strategies to encourage and support their engagement in activities. According to Tew, Jones, and Mikocka-Walus, as the course of IBD progresses, fatigue, anxiety, and depression tend to worsen, leading to a corresponding decrease in the PA levels of patients [44]. In addition, most participants (677/859) in the study indicated that IBD limited their ability to engage in PA, with influencing factors such as abdominal/joint pain (70%), fatigue/fatigue (69%), disease onset (63%), and increased urgency to use the toilet (61%). Furthermore, the PA levels of patients with CD were independently associated with depression, disease activity, and motor dysfunction. An online survey conducted by the Crohn's Disease and Colitis Association in the United Kingdom was used to assess exercise habits among the 918 participants with IBD [46]. The findings indicated that patients with IBD generally accepted PA, with a majority (72%) reporting positive feelings toward PA. However, 80% of the survey respondents indicated that they had to temporarily or permanently stop exercising at some point due to severe disease symptoms. IBD significantly hindered their ability to participate, enjoy, and derive benefits from PA. A semistructured interview study conducted in the Netherlands examined the role of PA in patients with IBD. The findings indicated that 86% of patients experienced positive effects from PA, such as improvement in overall health, quality of life, and self-image. However, symptoms experienced during the active phase of IBD often lead to postponement or discontinuation of PA [47].

## 5. Impact of PA on the Clinical Outcomes of Patients With IBD

**Figure 1:**
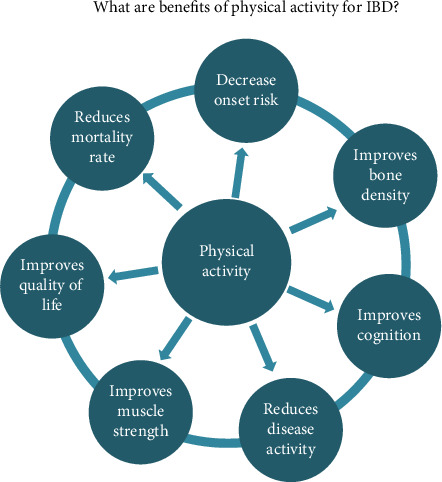
The impact of physical activity on IBD patients.

According to population-based evidence, PA can lower the risk of several chronic diseases, such as cancer and cardiovascular disease, and enhance patient prognosis [8, 48]. Nevertheless, the safety and potential health benefits of PA for patients with IBD remain unclear. Before recommending specific PA practices, it is essential to understand their impact on the clinical outcomes of patients with IBD, including disease activity, quality of life, and mortality. There are many benefits of PA for people with IBD (Figure 1). We also summarized the impact of PA on the clinical outcomes of IBD (Table 3).

### 5.1. PA and Disease Activity

Several studies have investigated the correlation between PA and disease activity, but the findings have been inconsistent. PA level may be associated with disease activity in patients with CD compared with those with UC. A cross-sectional survey [47] conducted in the Netherlands revealed a negative relationship between PA levels and disease activity in patients with CD. Patients with higher PA levels had lower disease activity. In contrast, no correlation was observed between PA levels and disease activity in patients with UC. A prospective cohort study conducted in the United States examined the relationship between PA levels and disease activity over 6 months in patients with IBD [49]. The findings revealed that patients with remission-stage CD with higher PA levels experienced a significant decrease in disease activity after 6 months (OR = 0.72, 95% CI: 0.55–0.94, *p* = 0.02). However, remission-stage UC patients with higher PA levels did not exhibit a significant decrease in disease activity after 6 months (OR = 0.78, 95% CI: 0.54–1.13, *p* = 0.18). Watanabe et al. [50] investigated the correlation between four types of PA (sedentary, standing, walking, and vigorous activity) and clinical remission in 327 patients with UC. No significant correlation was observed between mucosal healing and any type of PA and clinical remission in patients with UC. However, high vigorous activity levels and total daily METs may be negatively correlated with mucosal healing but not with clinical remission. In a randomized controlled study conducted by Seeger et al., 45 patients with CD in remission or mild activity were randomly assigned to a control group, endurance group, or muscle training group for 3 months, with PA intervention thrice weekly [51]. The findings indicated no significant change in disease activity between the two groups; however, both intervention groups reported improved quality of life. In summary, PA might help mitigate disease activity in patients with IBD, particularly patients with CD. Although the findings from various studies may vary, no negative effect of PA on disease activity has been found.

### 5.2. PA and Mortality Rate

Cucino and Sonnenberg [52] examined the mortality data of patients with IBD from 1991 to 1996 at the National Center for Health Statistics in the United States. They observed that after adjusting for gender and race, patients with IBD in occupations involving physical labor and agriculture (such as farmers and workers) had lower mortality rates compared with those in sedentary occupations involving indoor work (such as salespeople and secretaries). Lo et al. [53] used the Cox proportional hazards model to examine three large cohort data: Nurse Health Study (1986–2014), Nurse Health Study II (1991–2015), and Health Professional Follow-up Study (1986–2014). They observed that among 828 patients with IBD (363 CD and 465 UC), patients with UC with high PA levels had the lowest risk of all-cause mortality (HR = 0.14–0.41, *p* = 0.002). However, no significant correlation was observed in patients with CD.

### 5.3. PA and Quality of Life

Engaging in PA can enhance the quality of life for patients with IBD. A cross-sectional study conducted in South Korea investigated the correlation between PA levels and quality of life in patients with IBD [54]. The study included 158 patients with IBD and evaluated their PA levels using the IPAQ, the European Qol Five-Dimensional Questionnaire, and the European Quality of Life Visual Analog Scale. The findings indicated that higher PA levels were associated with improved quality of life, and improvement in leisure activities and nonsweating exercise was particularly associated with improved quality of life. In an intervention study, Loudon et al. [55] developed a 12-week walking plan for patients with sedentary remission or mild active CD. The exercise plan involved walking thrice weekly, and 12 patients completed it. The study revealed that after the intervention, patients exhibited significant improvements in the IBD stress index, quality of life score, BMI, and other outcome indicators. Similarly, Elsenbruch et al. [56] conducted an intervention for patients with UC in remission using a 10-week, 60-h structured training program. This program incorporated stress management training, moderate exercise, Mediterranean diet, behavioral skills, and self-care strategies. Quality of life was better in the intervention group than in the control group. However, no significant impact on clinical or physiological parameters was observed. Ng et al. [57] implemented a low-intensity walking program for 32 patients with CD in remission or mild activity. This program involved walking three times a week, with each session lasting 30 min for 3 months. Their findings indicated that patients who participated in the exercise program experienced a significant improvement in their quality of life, with no adverse effects observed on disease activity. Van Erp et al. [58] developed a 12-week intensive exercise program for patients with IBD in remission. This program included three sessions per week, each consisting of personalized high-intensity aerobic and progressive resistance training lasting for 1 h. Their study demonstrated that a personalized high-intensity exercise program can substantially improve fatigue, quality of life, and cardiopulmonary function in patients with IBD in remission. Recent studies [17, 18] have emphasized the bidirectional relationship between PA and IBD, advocating for the inclusion of PA in IBD management to improve patients' quality of life and reduce IBD-related complications. Our mixed study also showed that recreational PA was associated with improved patient-reported outcomes [63]. In summary, PA can potentially improve the quality of life of patients with IBD, although the PA schemes implemented in current studies vary widely. The approaches and safety considerations of PA are crucial for patients with IBD [64]. Future research can summarize the evidence from relevant studies to develop the best activity plan to guide the widespread adoption of PA.

### 5.4. PA and Body Composition

PA can cause changes in the body composition of patients with IBD, such as reductions in the levels of inflammatory biomarkers [65]. A previous study [59] used real-time detection of biochemical indicators in patients with IBD before and after PA to investigate the effects of PA on body composition. D'incà et al. [59] assessed the intestinal motility, permeability, and antioxidant levels of six remission-stage patients with CD and a healthy control population after PA. The participants were asked to engage in continuous activity for 1 h at a maximum oxygen consumption of 60%. The findings revealed that patients with CD had an increased cecal transit time after exercise compared with the control group. However, the difference was not significant, and the amount of urinary zinc was substantially increased. Another study [60] investigated the safety of moderate-intensity continuous and high-intensity intermittent activities in youth with CD. The study involved 30-min cycles with six cycles of 4 × 15 s at 50% and 100% peak mechanical power, respectively. Blood samples were collected at rest, midpoint during exercise, end of exercise, and 30 and 60 min after recovery to assess changes in inflammatory cells, cytokines, and growth factors. The findings indicated that neither activity led to exacerbation of acute inflammation. Seeger et al. [51] conducted moderate-intensity autologous weight and endurance training on patients with CD during the remission and mild activity phases, revealing that PA can enhance upper and lower limb strength as well as grip strength.

Furthermore, several studies have examined the long-term effects of PA in patients with IBD. In a randomized controlled trial conducted by Robinson et al. [61], 117 patients with CD were randomly assigned to either a control group or a low-intensity exercise program. Bone density of the hip joint and spine was measured using a dual-energy x-ray absorptiometer at baseline and after 12 months of exercise. After 12 months of follow-up, a significant relationship was observed between patient bone density and exercise frequency. Low-intensity exercise can enhance bone density in patients with CD, thereby reducing the risk of osteoporotic fractures. In a randomized controlled trial conducted by Jones et al. [62], 47 adults with CD in remission were randomly assigned to either the exercise or the control group. The exercise group underwent a 6-month resistance training program. At the end of the 6th month, the control group demonstrated substantial improvements in lumbar spine bone density, muscle strength, and physical performance compared with the exercise group.

## 6. Prospects for PA in IBD

The intensity and duration of exercise programs carried out vary from study to study. In a study conducted by Jones et al. [62], the exercise group received a 6-month combined impact and resistance training program, involving three, 60-min sessions per week and a gradual tapering of supervision to self-management. Moderate endurance exercise training and muscle training were performed for 30 min three times per week for 12 weeks in the study conducted by Seeger et al. [51]. In Ng's study [57], patients performed low-intensity walking at an interval of three times per week for a duration of 3 months, and each walking session lasted for 30 min. We can find that the exercise was mainly medium or low intensity, and the frequency was mostly three times a week, and each time was not more than 1 h.

In addition, the independent relationship between results and exercise frequency confirms the “training effect” of fully compliant patients [61]. However, some studies have unclear definitions of the duration and intensity of exercise interventions, as well as a lack of effective exercise supervision, which may lead to poor compliance among participants and dilute exercise effectiveness. Although current studies suggest the potential benefits of PA for IBD, the measurement of PA mostly relies on questionnaires, which may lead to recall bias. Wearable device–based accelerometers can more accurately measure PA.

With the aging population, the number of elderly people suffering from IBD is gradually increasing [66]. Elderly IBD patients face greater risks due to advanced age, more underlying diseases (such as hypertension and diabetes), and the use of hormones and immunosuppressants [67]. Some traditional Chinese medicine exercise rehabilitation programs, such as Tai Chi [68] and Ba Duan Jin [69], may be more suitable for elderly IBD patients, but there are currently no relevant reports among IBD patients. Resolving the above issues will help unlock greater power for PA in IBD.

Future study needs to explore the best type of exercise and the most appropriate exercise time from an evidence-based perspective, build an exercise program suitable for IBD patients of different ages and disease degrees, and finally verify the effectiveness of the exercise program through randomized controlled trials.

## 7. Limitations

This review has several limitations that deserve transparent discussion. Some studies have revealed the potential benefits of exercise for people with IBD; however, exercise programs vary from study to study, and our review focused on exercise effects without summarizing the characteristics of these exercise intervention programs. It is still necessary to sort out these exercise programs and explore the optimal type of exercise for people with IBD in the future. In addition, our review did not summarize the differences in the impact of exercise on different age groups, which is not conducive to developing personalized exercise prescriptions.

## 8. Conclusion

Patients with IBD have insufficient PA levels, and the correlation between PA and the incidence and disease activity of IBD remains controversial. Nevertheless, PA has demonstrated beneficial effects on clinical outcomes, particularly in reducing mortality, enhancing quality of life, and improving body composition. Future research should investigate various approaches, intensities, and volumes to determine the optimal activity plan for patients with IBD. In clinical practice, patients must undergo exercise health screenings and risk evaluations, considering patient goals, disease status, living conditions, and other relevant factors to tailor personalized exercise prescriptions for each patient.

## Figures and Tables

**Table 1:** Definition and examples of different intensity exercises.

**Exercise intensity**	**Maximal heart rate (%pred)**	**Metabolic equivalent (MET)**	**Examples**
Sedentary behavior	< 40	< 1.5	Lying or sitting
Low-intensity PA	71–85	1.5–2.9	Walking, laundry, and cooking
Moderate-intensity PA	61–70	3–5.9	Brisk walking, heavy cleaning work, and dancing
Vigorous-intensity PA	40–60	> 6	Running, doing heavy agricultural work, and kicking

**Table 2:** Researches on the impact of physical activity on the onset of inflammatory bowel disease.

**Author, year**	**Design**	**Study population**	**Study findings**
Sonnenberg, 1990 [33]	Observational study	7594 CD; 4420 UC	Sedentary and indoor occupations seem to be associated with a higher risk of IBD; exercising outdoors seems to be a protective working condition
Bøggild, Tüchsen, and Orhede, 1996 [34]	Cohort study	2,273,872 participants	Compared to occupations without sedentary work, occupations with predominantly sedentary work had a standardized hospitalization ratio of 125 (95% CI: 116.9–133.1)
Persson et al., 1993 [35]	Case–control study	152 CD; 145 UC; 305 controls	The relative risk of CD was inversely related to regular PA and estimated at 0.6 (95% CI: 0.4–0.9) and 0.5 (95% CI: 0.3–0.9) for weekly and daily exercise, respectively
Hlavaty et al., 2013 [36]	Case–control study	190 CD; 148 UC; 355 controls	CD associated with less than two childhood sporting activities weekly (OR = 2.7, 95% CI 1.5–5.0; *p* < 0.001); UC associated with less than two sporting weekly activities in childhood (OR = 2.0, 95% CI 1.1–3.5, *p* = 0.02)
Agrawal et al., 2021 [37]	Interview survey	60,155 participants	IBD (*n* = 786) was associated with insufficient PA (OR = 1.38, 95% CI 1.16–1.66)
Klein et al., 1998 [38]	Case–control study	55 UC; 33 CD; 76 matched population and 68 clinic controls	IBD patients had low of PA during the preillness period (*p* < 0.001) as compared to controls
Khalili et al., 2013 [39]	Cohort study	194,711 participants	The risk of CD was inversely associated with PA (*p* = 0.02). Compared with women in the lowest fifth of PA, the HR among women in the highest fifth of PA was 0.64 (95% CI: 0.44–0.94). Active women with at least 27 MET-h/week of PA had a 44% reduction (HR: 0.56, 95% CI 0.37–0.84) in risk of developing CD compared with sedentary women with < 3 MET-h/week. PA was not associated with risk of UC (*p* = 0.46)
Chan et al., 2013 [40]	Cohort study	300,724 participants	No significant correlation was observed between PA and UC (*p* = 0.97) or CD (*p* = 0.42)
Halfvarson et al., 2006 [41]	Co–twin control study	317 twin pairs	There is no significant difference in PA between patients with IBD before diagnosis and their twins

Abbreviations: 95% CI: 95% confidence interval; CD: Crohn's disease; HR: hazard ratio; IBD: inflammatory bowel disease; PA: physical activity.

**Table 3:** Summary of the impact of physical activity on clinical outcomes of inflammatory bowel disease.

**Author, year**	**Design**	**Study population**	**Study findings**
*Physical activity and disease activity*			
Lamers et al., 2021 [47]	Cross-sectional study	176 CD; 162 UC	In CD, the total PA score was inversely associated with disease activity, after adjustment for confounders (*β* = −0.375; *p* = 0.013); no association between PA and disease activity was found in UC
Jones et al., 2015 [49]	Cohort study	1308 CD; 549 UC	Patients with remission-stage CD with higher PA levels experienced a significant decrease in disease activity after 6 months (OR = 0.72, 95% CI 0.55–0.94, *p* = 0.02). Remission-stage UC patients with higher PA levels did not exhibit a significant decrease in disease activity after 6 months (OR = 0.78, 95% CI 0.54–1.13, *p* = 0.18)
Watanabe et al., 2021 [50]	Cross-sectional study	327 UC	Plentiful strenuous activity (OR = 0.24, 95% CI 0.07–0.62; *p* = 0.008) and a very high daily MET total (OR = 0.37, 95% CI 0.16–0.80; *p* = 0.010) were both significantly inversely associated with complete mucosal healing. However, no association between PA and clinical remission was found
Seeger et al., 2020 [51]	RCT	Endurance group: 17 CD; muscle group: 15 CD; control group: 13 CD	Exercise has no effect on disease activity (all *p* > 0.05)

*Physical activity and mortality rate*			
Cucino and Sonnenberg, 2001 [52]	Observational study	13,539,945 participants	IBD mortality is low in occupations associated with manual work and farming (PMR = 39–64, *p* < 0.05) and relatively high in sedentary occupations associated with indoor work (PMR = 177–122)
Lo et al., 2021 [53]	Cohort study	363 CD; 465 UC	Patients with UC with high PA levels had the lowest risk of all-cause mortality (HR = 0.14–0.41, *p* = 0.002). However, no significant correlation was observed in patients with CD

*Physical activity and quality of life*			
Kim et al., 2021 [54]	Observational study	158 IBD	Increased PA levels were associated with improved QOL in patients with IBD. More leisure activity and non–sweat-inducing exercise were associated with improved QOL in patients with IBD
Loudon et al., 1999 [55]	Experimental study	12 CD	12-week walking program improved the inflammatory bowel disease stress index, the inflammatory bowel disease quality of life score, the Harvey and Bradshaw Simple Index, the Canadian Aerobic Fitness Test, VO_2_ max, and body mass index (all *p* < 0.05)
Elsenbruch et al., 2005 [56]	RCT	Intervention group: 15 CD; usual-care control group: 15 CD	Mind–body therapy may improve quality of life in patients with UC in remission, while no effects of therapy on clinical or physiological parameters were found
Ng et al., 2007 [57]	RCT	Exercise group: 16 CD; control group: 16 CD	Patients in the exercise group experienced a statistically significant (*p* < 0.05) improvement in quality of life in all 3 of the outcome measurement questionnaires with no detrimental effects in terms of disease activity
Van Erp, 2021 [58]	Experimental study	25 IBD	A personalized, intensive exercise program can lead to significant improvement of fatigue, HRQoL, and cardiorespiratory fitness in patients with quiescent IBD and severe fatigue

*Physical activity and body composition*			
D'incà et al., 1999 [59]	Experimental study	6 CD; 6 controls	Orocaecal transit time increased after exercise in CD patients (72 ± 30 min vs. 100 ± 34 min) with no significant difference from controls (77 ± 20 min vs. 83 ± 23 min). Zinc urinary output significantly increased after exercise in CD patients and remained unchanged in control subjects
Ploeger et al., 2012 [60]	Experimental study	15 CD; 15 controls	Both moderate-intensity continuous exercise and high-intensity intermittent exercise increased immune cells and GH and decreased IGF-I. Moderate-intensity exercise induced a greater increase in leukocytes (*p* < 0.05), neutrophils (*p* < 0.05), lymphocytes (*p* < 0.001), monocytes (*p* < 0.05), IL-6 (*p* < 0.05), IL-17 (*p* < 0.05), and GH (*p* < 0.05) and a similar decrease in IGF-I, compared with high-intensity exercise
Seeger et al., 2020 [51]	RCT	Endurance group: 17 CD; muscle group: 15 CD; control group: 13 CD	In both exercise groups, the maximal and average strength in the upper and lower extremities increased significantly (vs. control, all *p* < 0.04). In the endurance group, emotional function was significantly improved (*p* = 0.03)
Robinson et al.,1998 [61]	RCT	Low-impact exercise group: 54 CD; control group: 53 CD	Compared with controls, the bone density of the greater trochanter significantly increased (difference in means, 4.67; 95% Cl 0.86–8.48; *p* = 0.02)
Jones et al., 2020 [62]	RCT	Exercise group: 23 CD; control group: 24 CD	At 6 months, BMD values were superior in the exercise group with statistical significance at the lumbar spine (adjusted mean difference 0.036 g/cm^2^, 95% CI 0.024–0.048; *p* < 0.001). The exercise group also had superior values for all muscle function outcomes (*p* < 0.001)

Abbreviations: BMD: bone mineral density; PMR: proportional mortality ratio; QOL: quality of life; RCT: randomized controlled study.

## Data Availability

The authors have nothing to report.
